# A Data-Driven Framework for Clinical Decision Support Systems in Positive Airway Pressure and Oxygen Titration

**DOI:** 10.3390/jcm13030757

**Published:** 2024-01-28

**Authors:** Artis Svaža, Dāvis Freimanis, Dana Zariņa, Pavels Osipovs, Svjatoslavs Kistkins, Vitālijs Ankudovičs, Olegs Sabeļnikovs, Valdis Pīrāgs, Yuriy Chizhov, Dmitrijs Bliznuks

**Affiliations:** 1 Sleep Disorder Clinic, LV-1002 Riga, Latvia; 2 Department of Internal Medicine, Pauls Stradiņš Clinical University Hospital, LV-1002 Riga, Latvia; 3 Institute of Applied Computer Systems, Riga Technical University, LV-1046 Riga, Latvia; 4 Department of Clinical Skills and Medical Technology, Riga Stradiņš University, LV-1007 Riga, Latvia

**Keywords:** obstructive sleep apnea, PAP titration, hypoxia, hypercapnia, Markov decision processes

## Abstract

Background: Current obstructive sleep apnea treatment relies on manual PAP titration, but it has limitations. Complex interactions during titration and variations in SpO_2_ data accuracy pose challenges. Patients with co-occurring chronic hypercapnia may require precise oxygen titration. To address these issues, we propose a Clinical Decision Support System using Markov decision processes. Methods: This study, compliant with data protection laws, focused on adults with OSA-induced hypoxemia utilizing supplemental oxygen and CPAP/BiPAP therapy. PAP titration, conducted over one night, involved vigilant monitoring of vital signs and physiological parameters. Adjustments to CPAP pressure, potential BiLevel transitions, and supplemental oxygen were precisely guided by patient metrics. Markov decision processes outlined three treatment actions for disorder management, incorporating expert medical insights. Results: In our study involving 14 OSA patients (average age: 63 years, 27% females, BMI 41 kg m^−2^), significant improvements were observed in key health parameters after manual titration. The initial AHI of 61.8 events per hour significantly decreased to an average of 18.0 events per hour after PAP and oxygen titration (*p* < 0.0001), indicating a substantial reduction in sleep-disordered breathing severity. Concurrently, SpO_2_ levels increased significantly from an average of 79.7% before titration to 89.1% after titration (*p* < 0.0003). Pearson correlation coefficients demonstrated aggravation of hypercapnia in 50% of patients (*N* = 5) with initial pCO_2_ < 55 mmHg during the increase in CPAP pressure. However, transitioning to BiPAP exhibited a reduction in pCO_2_ levels, showcasing its efficacy in addressing hypercapnia. Simultaneously, BiPAP therapy correlated with a substantial increase in SpO_2_, underscoring its positive impact on oxygenation in OSA patients. Markov Decision Process analysis demonstrated realistic patient behavior during stable night conditions, emphasizing minimal apnea and good toleration to high CPAP pressure. Conclusions: The development of a framework for Markov decision processes of PAP and oxygen titration algorithms holds promise for providing algorithms for improving pCO_2_ and SpO_2_ values. While challenges remain, including the need for high-quality data, the potential benefits in terms of patient management and care optimization are substantial, and this approach represents an exciting frontier in the realm of telemedicine and respiratory healthcare.

## 1. Introduction

According to the American Academy of Sleep Medicine (AASM) clinical guidelines, manual titration of positive airway pressure during attended polysomnography is the current standard for the selection of the optimal patient therapeutic pressure for patients with obstructive sleep apnea. Continuous positive airway pressure (CPAP) and bi-level positive airway pressure (BiPAP) are the two forms of PAP that are manually titrated during a PSG to determine the optimal amount of pressure for subsequent nightly usage. In pursuing effective and well-tolerated airway pressure therapy, patients often undergo PAP titration procedure for diagnostic measurements [1]. This essential step aims to determine the appropriate air pressure required to maintain an open airway during sleep. However, the current strategies for PAP titration have certain disadvantages. Manual adjustments of parameters, e.g., pressure and oxygen fraction, can be challenging due to complex interactions with other NIV parameters like tidal volume and PEEP (Positive End-Expiratory Pressure) [2].

Furthermore, the accuracy of obtained SpO_2_ data may vary, potentially introducing errors in the titration process [3]. Some individuals may necessitate long-term oxygen therapy, even when using PAP devices [4,5]. A common co-occurring condition in OSA is chronic hypercapnia, which may further necessitate oxygen supplementation. Chronic hypercapnia can have adverse effects on respiratory and cardiovascular systems, making its management a crucial aspect of OSA treatment. Additional oxygen supplementation in positive airway pressure treatment increases blood oxygen saturation, but it may also lead to bradypnea in patients with chronic hypercapnia, exacerbating their condition [6]. This emphasizes the importance of precise oxygen titration techniques, especially when addressing chronic hypercapnia co-occurring with OSA.

Recognizing the disadvantages of manual adjustments, this study tackles the development of a Clinical Decision Support System (CDSS) for time efficiency in the titration of airway pressure and additional oxygen supply. Automation and expert-based control systems are crucial components, offering the potential for enhanced treatment adaptability and effectiveness while minimizing patient discomfort and time-consuming healthcare provider burden. The application of Markov decision processes (MDP) as a CDSS presents a significant yet intricate challenge, introducing innovative possibilities along with complex issues. MDPs provide a framework for modeling sequential decision making under uncertainty, allowing decision makers to maximize the probability of reaching desired states [7].

To address limitations of manual titration and enhance the precision of OSA treatment, the integration of Clinical Decision Support Systems (CDSS), based on Markov decision processes, for automated PAP and oxygen titration has been explored in this study to describe the potential markers for the CPAP transition to BiPAP.

## 2. Materials and Methods

This descriptive observatory study was conducted and marketed in conformity with all regulatory enactments regulating data protection in the Republic of Latvia, including the Law on the Processing of Personal Data (protocol number E1.1.1.1/21/A/082, clinicaltrials.gov: NCT06090149). The patient population consisted of a case series of adults with OSA-induced hypoxemia who used additional oxygen supply and CPAP/BiPAP therapy. Patients included in the study were initially identified by reviewing medical records at Pauls Stradins Clinical University Hospital (PSCUH) for hospital patient medical cards. The study’s inclusion criteria encompassed individuals aged 18 and above who had OSA with specified criteria such as AHI values, documented symptoms, and associated health issues, as well as Chronic Obstructive Pulmonary Disease (COPD) with documented hypoxemia without additional oxygen support. Additionally, conditions involving the overlap between COPD and OSA or between pulmonary hypertension and obstructive sleep apnea were considered. On the other hand, the exclusion criteria involved individuals with COPD and OSA lacking documented hypoxemia, those under 18 years old, those requiring permanent replacement therapy for another organ, individuals contraindicated in non-invasive pulmonary ventilation, those refusing study participation, those with cognitive impairment hindering research completion, individuals with claustrophobia, pregnant individuals, and those with severe heart failure or recent cardiovascular events according to New York Heart Association functional class III–IV within the month preceding study inclusion.

During the study, patients were subjected to PAP titration while receiving supplemental oxygen through an oxygen concentrator. The research took place over one night, spanning from 22:00 to 8:00, covering a 10 h duration. A sleep technician meticulously observed a variety of vital signs and physiological parameters including monitoring respiratory flow, thoracic and abdominal movements, body position, respiratory rate (RR), heart rate (HR), blood oxygen saturation (SpO_2_), and levels of carbon dioxide (CO_2_) during transcutaneous measurements. The study also involved the collection of significant data, including the apnea–hypopnea index (AHI), and sleep architecture. Throughout the night, the sleep technician observed the patient’s condition, ensuring precise monitoring of all relevant parameters. This included the vital task of fine-tuning CPAP/BiLEVEL settings, adapting unit pressures, and controlling oxygen delivery as needed. These adjustments were carefully guided by the patient’s thoracic and abdominal movements, airflow patterns, blood SpO_2_ levels, and pCO_2_ concentrations. To facilitate this monitoring and care, patients were placed in a dedicated room equipped with monitoring and treatment equipment. The physician assistant conducted their observations and managed patient care from a separate room, utilizing remote control to make necessary adjustments.

PAP titration and data acquisition protocol: The CPAP pressure underwent a gradual adjustment to alleviate or eliminate apnea episodes. In cases where patients experienced tolerance issues, the PEEP level was lowered or switched to a BiLevel treatment. Supplementary oxygen was introduced based on the patient’s SpO_2_ levels, with a target of maintaining SpO_2_ levels of at least 89%. Continuous monitoring of pCO_2_ levels was carried out, and if there was a 5-unit increase in pCO_2_ or if SpO_2_ levels exceeded 92%, the oxygen flow was tapered down. The data acquisition process involved using the Lowenstein medical BiLevel Prisma 25ST device (software v5.22), which offered various modes such as CPAP, APAP, and BiLevel. This device also facilitated online monitoring of pressure, tidal volume, and mask leaks. Additionally, pCO_2_ levels were monitored using the Sentec device, which seamlessly integrated with the Lowenstein medical diagnostics report, and all the parameters were displayed in real-time on the screen. To complement the data collection, supplemental oxygen was administered through the Devilbiss 525 KS oxygen concentrator. When necessary, a physician’s assistant manually conducted additional data acquisition, particularly while making adjustments to the PAP settings and when adding or removing oxygen from the treatment.

Diagnostic prediction task: The treatment plan for patients is based on three key conditions: apnea, pCO_2_ levels, and changes in SpO_2_ levels. The target conditions for patients are absence of apnea, SpO_2_ levels above 92%, and stable pCO_2_ levels. The ideal progression for patients involves eliminating apnea and achieving target SpO_2_ levels without an increase in pCO_2_, while maintaining the lowest possible CPAP/BPAP pressure. The controllable parameters in this treatment are the CPAP/BPAP pressure and the flow of oxygen.

**Figure 1:**
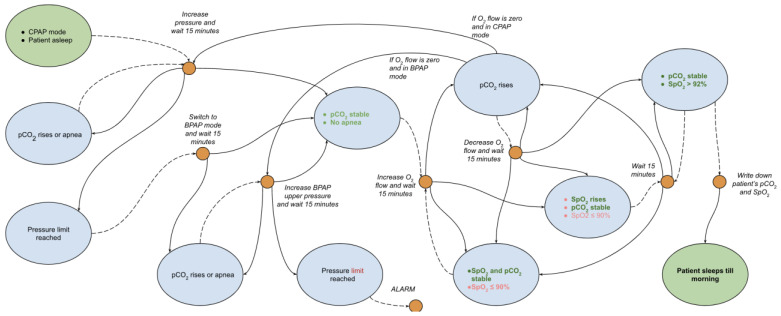
Expert knowledge-based model for PAP and oxygen titration.

These controllable factors are interdependent and do not often change in the ideal way described above. As a result, the diagnostic system must be capable of determining the appropriate treatment action for all possible changes in these conditions. The Markov decision process, as illustrated in Figure 1, accounts for potential worsening of conditions and outlines corresponding treatment actions. Typically, patients will spend most of their time adjusting the oxygen flow and CPAP/BPAP pressure to balance between increasing their SpO_2_ and decreasing their pCO_2_ levels.

## 3. Results

### 3.1. Patient Cohort Description

The final patient cohort comprised 14 OSA patients with sleep-disordered breathing evaluated in the Sleep Clinics between November 2022 and August 2023 (Supplementary 1, Table S1). The cohort was selected based on PSG or strong clinical evidence of significant improvement in OSA following a single-site operative intervention. The average age was 63 years (range 50–79 years, standard deviation [SD] 8 years). Of the study group, 27% were females (N = 4). The average body mass index (BMI) was 41 kg m^−2^ (range 23–55 kg m^−2^, SD 9 kg m^−2^). The average AHI of the PSGs (one patient contributed two separate PSGs at clinically discrete time points) included in our training and testing datasets was 61.8 events per hour (range 21.4–109.2 h, SD 29.1 h). The average initial blood oxygen saturation was 79.7% (range 67.1–90%, SD 7.72%). The average initial pCO_2_ was 53 mmHg (range 39–63 mmHg, SD 8 mmHg). In total, 2 patients were normocapnic (<45 mmHg), while 12 patients were hypercapnic (9 patients had initial pCO_2_ level below 55 mmHg, while 4 had above 55 mmHg).

### 3.2. Results of the PAP and Oxygen Titration

The results of the PAP and oxygen titration are summarized in Table 1 and revealed statistically significant improvements in SpO_2_.

The average blood oxygen saturation increased to 89.1% (range 79.2–98.6%, SD 5.0%), reflecting a substantial enhancement in oxygen levels during sleep. The average delta values of SpO_2_ were 9.4% (range 0.4–21.9%, SD 6.25%). The median delta values of SpO_2_ were 8.1%, IQR 8.5%.

Additionally, the AHI index demonstrated a statistically significant reduction, with an average decrease to 18.0 events per hour (range 1.3–46.8 events/h, SD 13.9), highlighting a substantial amelioration in sleep-disordered breathing. The average Delta values of AHI were 48.0 (range −18.6–89.9 events/h, SD 33.1), while the median values were 53.5 events/h, IQR 46.45 events/h. These findings underscore the positive impact of the single-site operative intervention on key physiological parameters in our patient cohort. *t*-test shows *p* < 0.0001.

In the case of pCO_2_ levels, there were no statistically significant changes in pCO_2_ before and after the PAP titration (average 53 mmHg, range 43–59 mmHg, SD 5 mmHg). The average delta values of pCO_2_ were 1 mmHg (range 6–9 mmHg, SD 5 mmHg). 

### 3.3. Pearson Correlation—Pressure/pCO_2_/SpO_2_. Indications to Proceed to BiPAP Therapy

Table 2 examines the Pearson correlation coefficients for pCO_2_ and SpO_2_ in relation to CPAP and IPAP pressure values. Normocapnic patients (N = 2) with SpO_2_ >88% at the beginning of the PAP titration remained normocapnic within the treatment. Notably, 50% of the patients whose initial pCO_2_ was below 55 mmHg (N = 5) during CPAP intervention exhibited a positive Pearson correlation coefficient for pCO_2_. Conversely, those with an initial pCO_2_ above 55 mmHg (N = 4) did not correlate strongly with pCO_2_. When CPAP therapy was transitioned to BiPAP therapy (N = 7), there was a strongly correlated decrease in pCO_2_ corresponding to the increase in IPAP pressure in all patients.

A comparative analysis between CPAP and BiPAP therapies revealed a higher amount of significantly stronger correlations in the increase in SpO_2_ with BiPAP therapy. Within the BiPAP titration, the increase in oxygenation was strongly correlated in 57% of patients (N = 4), while a strong positive correlation on the CPAP treatment was observed in only 7% of patients (N = 1). A strong negative correlation was observed in one patient who received CPAP therapy. Notably, for those patients whose pCO_2_ > 55 mmHg, there was no strong correlation in an increase in SpO_2_ within CPAP treatment, while BiPAP therapy showed a strong correlation in 50% of cases (N = 2). 

### 3.4. Markov Decision Process

In order to describe the Markov decision process, two patients were selected that had more than one hour of intervention sleep and had additional supplementary oxygen therapy. Supplementary Materials 1, Table S2, holds screenshots from polysomnography application, where all parameters can be overseen. Table S2a shows the MDP’s first step, where CPAP pressure is raised until apnea episodes disappear (12 mmH_2_O). Apnea episodes are shown as pink rectangles. The next decision step is where pCO_2_ is stable and oxygen is added. Table S2b shows that pCO_2_ stabilizes at 60 hmmHg and even decreases to 55 mmHg, and then, 1 L/min of oxygen is added, promoting an increase in SpO_2_. After a while, the next MDP step can be seen (Table S2c) where pCO_2_ is rising from 55 to 60 mmHg after an increase in oxygen supplementation to 2 L/min of oxygen supplementation. After decreasing oxygen to 1 L/min, pCO_2_ drops to 53. At the same time, SpO_2_ were rising during O_2_ of 2 L/min, reaching 96% and after switching to 1 L/min, drops to 92%, which is acceptable. After a while, the patient woke up at 5 am and was mostly stabilized; nevertheless, pCO_2_ could be better. As a result, the MDP shows realistic behavior on the patients that were stable in the night with minimal amount of apnea and good toleration of high CPAP pressure.

## 4. Discussion

The average overall increase in SpO_2_ to 89.1% reflects a substantial improvement, with noteworthy average delta values of SpO_2_ of 9.4%. The consistency of this improvement is further emphasized by the narrow standard deviation (SD) of 5.0%, suggesting a reliable and predictable response to manual PAP therapy across the patient cohort. The results, however, show the stronger correlation in oxygen support on the higher IPAP values, while the increase in the CPAP pressure did not strongly correlate with the increase. The main explanation might be the impact of the higher CPAP pressure on the exhale phase as well as the impact of the apnea events that may impact the fluctuation [8]. 

According to the American Academy of Sleep Medicine (AASM) guidelines for conducting manual PAP therapy, a BiPAP device may be used when a patient demonstrates difficulty acclimating to high airway pressure during the expiration phase of breathing or requires ventilatory support [1]. The indications for transitioning to BiPAP therapy include patient intolerance to a CPAP therapy, persistent symptoms like excessive daytime sleepiness or snoring despite CPAP usage, and the observation of residual obstructive events at a CPAP pressure of ≥15 cm H_2_O. When evaluating suspected etiologies for these issues, if retitration with the same modality proves unsuccessful, the AASM recommends considering bilevel positive airway pressure (BPAP) therapy, particularly in the spontaneous mode. However, it is essential to note that the decision to switch to BiPAP is predominantly influenced by apnea events, with pCO_2_ and SpO_2_ monitoring playing a secondary role. It is worth mentioning that patients with hypoxia on CPAP therapy may require additional oxygen therapy, thus the speculation regarding the initiation of the O_2_ still persists. For instance, the administration of supplemental oxygen may exacerbate carbon dioxide retention, particularly in cases of type 2 respiratory failure. Notably, elevated oxygen concentrations may lead to a reduction in the respiratory drive, subsequently diminishing the ventilatory response. This diminished drive can impede the efficient elimination of CO_2_, potentially exacerbating hypercapnia [6].

It is crucial to observe the subgroup of patients whose initial pCO_2_ levels were below 55 mmHg during CPAP intervention. Among this cohort (N = 5), a positive Pearson correlation coefficient for pCO_2_ was evident. This implies that, for a subset of patients with pCO_2_ < 55 mmHg levels, there was a discernible positive association between pCO_2_ and CPAP pressure values. Such a relationship may be also indicative of a physiological response to higher positive airway pressure, possibly implicating a greater challenge in exhaling effectively under these conditions. Conversely, patients with an initial pCO_2_ above 55 mmHg (N = 4) did not exhibit a strong correlation between pCO_2_ and CPAP pressure values. This lack of positive and negative correlation suggests that for individuals with higher baseline pCO_2_ levels, the impact of CPAP pressure on pCO_2_ dynamics may be less pronounced. It is plausible that these patients have a different respiratory response or compensatory mechanisms that mitigate the influence of positive airway pressure on pCO_2_ levels. Transitioning from CPAP therapy to bilevel positive airway pressure therapy revealed a consistent and strongly correlated decrease in pCO_2_ as IPAP pressure increased across all patients (N = 7). This finding implies that as the inspiratory positive airway pressure intensified, there was a corresponding reduction in pCO_2_ levels. BPAP allows the sleep technologist to separately adjust inspiratory and/or expiratory pressures during the polysomnogram to maintain the patency of the airway and provide ventilatory support. According to AASM guidelines, when transitioning from continuous positive airway pressure (CPAP) to BiPAP, the minimum starting expiratory positive airway pressure (EPAP) should be set at 4 cm H_2_O or the CPAP level at which obstructive apneas were eliminated. An optimal minimum inspiratory positive airway pressure (IPAP)–EPAP differential is 4 cmH_2_O and an optimal maximum IPAP–EPAP differential is 10 cmH_2_O_2_ [1]. This inverse relationship between IPAP pressure and pCO_2_ suggests that, in the context of BiPAP therapy, higher inspiratory pressures facilitate a more efficient removal of carbon dioxide [9,10].

The statistically significant improvements in SpO_2_ and AHI following PAP and oxygen titration underscore the effectiveness of the manual PAP therapy in enhancing blood oxygen saturation during sleep. The reduction in the Apnea–Hypopnea index from an average of 61.8 events per hour before titration to 18.0 events per hour after titration demonstrates a statistically significant manual PAP titration efficiency. Similar results of the manual titration were obtained in other studies [11]. However, it is time consuming to manually define the pressure support and the decision to switch the therapy from CPAP to BiPAP [12]. Adjusting PAP parameters manually during the night presents several challenges, including the overload of sleep technologists. Moreover, this manual approach may increase the risk of human error in documentation and decision making [13]. The differences between automatic PAP titration and manual titration may come from how parameters such as apnea, hypopnea, and snoring are defined, as well as the use of different recording methods [2]. 

Several previous publications have raised the clinical problem of poor adherence of CPAP in OSA and telemedicine could be one of the solutions [14]. 

Within this study, we have also observed the Markov decision processes. In this study, we delved into the utilization of MDPs within the context of a patient-centered and remote healthcare system. The significance of MDPs in this modern healthcare approach is increasingly recognized. MDPs are mathematical frameworks that can calculate the likelihood of transitioning between various states, defined by observing a large set of real condition changes. This methodology is particularly innovative in the management of positive airway pressure (PAP) and oxygen titration, where it can make decisions taking into account the evolving respiratory health of patients, thereby facilitating informed and timely treatment decisions. Using different system evaluation methods can help identify system defects and correct them in further research [15].

The integration of MDPs into these algorithms allows for the consideration of the fluid nature of respiratory ailments. By doing so, treatment parameters can be adjusted based on the patient’s current health status and their medical history, ensuring a more tailored approach to care. The adaptability of these algorithms, underpinned by MDPs, marks a significant breakthrough in respiratory healthcare management. It enables healthcare providers to offer more accurate care remotely that not only brings the decision but is able to explain it. This approach aligns with the evolving landscape of healthcare, where personalized treatment and remote patient management are becoming increasingly important. The use of MDPs in this regard not only enhances patient outcomes but also streamlines the healthcare process, making it more efficient and responsive to patient needs.

Limitation: However, applying MDPs necessitates gathering not only primary data related to treatment but also extensive meta-data or detailed data that are typically not collected during treatment. For example, detailed records of a patient’s restless sleep progression are often not documented. Additionally, the automatic construction of a Markov transition graph implies repeatedly taking all possible actions for each state, known as environmental exploration. Of course, in medical scenarios, such an approach cannot be applied to patients. Instead, the transition matrix is constructed using existing treatment records, deviating from the idea of complete environmental exploration. Even with such an approach, there is a risk of getting an imprecise model. For example, in the study [16], it is mentioned that the estimates of rewards and transition dynamics used to parameterize the MDPs are often imprecise and lead the DM to make decisions that do not perform well with respect to the true system. The imprecision in the estimates arises due to these values being typically obtained from observational data or from multiple external sources [16]. When the policy found via an optimization process using the estimates is evaluated under the true parameters, the performance can be much worse than anticipated [17]. Another approach for building or testing policy is using simulations [18] that are possible in certain scenarios.

Markov decision process-based telemedical algorithms for patient treatment begin with creating an initial graph using expert medical knowledge. This method addresses the challenges of limited data collection and incomplete environmental exploration. The crucial next step is refining this graph (Figure 1) by testing it against historical patient treatment records to develop a transition between states, a complex yet essential phase for building a functional Markov decision process. This approach, while intricate in implementation, is vital for overcoming inherent limitations similar to those faced by neural networks in predicting oxygen and pCO_2_ fluctuations. It requires extensive, accurate data to create effective algorithms, a challenging but critical aspect of ensuring patient data quality and completeness. The optimism for this methodology stems from the demonstrated successes of machine learning in healthcare, particularly in enhancing patient outcomes in smaller, simulation-based studies, whereas neural networks require a much larger dataset to achieve acceptable treatment accuracy. The future of this development lies in analyzing a larger pool of patient records, which will help in establishing statistical probabilities of state changes for more accurate predictions and individualized patient modeling, marking a significant step towards personalized healthcare through technology.

Despite the acknowledged challenges, Markov process-based algorithms have the potential to be additionally trained to specific patients’ respiratory conditions, using additional hidden layers. This may potentially improve patient outcomes and enhance the quality of care in the field of respiratory medicine.

In conclusion, the development of a framework for Markov decision processes of PAP and oxygen titration algorithms holds promise for providing algorithms for deviation in CO_2_ and SpO_2_ markers. While challenges remain, including the need for high-quality data, the potential benefits in terms of patient management and care optimization are substantial, and this approach represents an exciting frontier in the realm of telemedicine and respiratory healthcare.

## Figures and Tables

**Table 1:** Titration results.

Parameter	Value	SD
Age, years	63.0	8.0
BMI	41.0	9.0
F:M	27%	
AHI before titration	61.8	7.3
AHI after titration	18.0	13.9
SpO_2_ before titration (%)	79.7	7.7
SpO_2_ after titration (%)	89.1	5.0
*t*-test (AHI)	*p* < 0.0001	
*t*-test (SpO_2_)	*p* < 0.0003	

**Table 2:** pCO_2_ and SpO_2_ Pearson correlation coefficients according to titration choice and PAP pressure.

Patient	Hypercapnia	Before (SpO_2_ >88%)	After (SpO_2_ >88%)	Transition to BiPAP	Pearson Correlation Coefficient for pCO_2_		Pearson Correlation Coefficient for SpO_2_	
					CPAP	BiPAP	CPAP	BiPAP
1	<55 mmHg	No	No	Yes	0.77	−0.67	−0.69	0.48
2	>55 mmHg	Yes	Yes	Yes	−0.29	−0.60	0.30	0.52
3	>55 mmHg	No	No	Yes	0.09	−0.72	−0.25	0.02
4	No	No	Yes	No	0.61		0.22	
5	<55 mmHg	No	Yes	No	0.22		0.40	
6	<55 mmHg	No	Yes	Yes	0.75	−0.68	−0.43	0.63
7	>55 mmHg	No	No	Yes	−0.08	−0.72	−0.07	0.02
8	<55 mmHg	No	Yes	No	−0.58		−0.16	
9	>55 mmHg	Yes	Yes	Yes	0.01	−0.44	0.30	0.50
10	<55 mmHg	No	No	No	0.08		−0.16	
11	No	No	Yes	No	0.78		0.69	
12	<55 mmHg	No	No	No	−0.21		0.40	
13	<55 mmHg	No	Yes	No	0.66		0.02	
14	<55 mmHg	No	Yes	Yes	0.14	−0.77	0.41	0.84

## Data Availability

The original contributions presented in the study are included in the article, further inquiries can be directed to the corresponding authors.

## Supplementary Materials

The following supporting information can be downloaded at: https://www.mdpi.com/article/10.3390/jcm13030757/s1, Table S1: Patients’ description, Table S2: Patient’s parameters during titration.
