# Supplementary material for: A Data-Driven Framework for Clinical Decision Support Systems in Positive Airway Pressure and Oxygen Titration
Source: J Clin Med. 2024 Jan 28;13(3):757. doi: 10.3390/jcm13030757 (PMC10856483; doi:10.3390/jcm13030757)
Supplement: Supplementary file 1 [file jcm-13-00757-s001.zip › jcm-2791782-supplementary.pdf]

Table S1. Patients' description

Patient	Sex	Height, cm	Weight, kg	Age, y	BMI	AHI (initial)	AHI (after)	SpO2 (initial)	SpO2 (after)	Diagnosis
1	M	175	130	65	42	88	**31**	80	**86,4**	Severe OSA, Type 2 Respiratory Failure, Grade 3 Obesity.
2	M	148	51	75	23	21,4	**40**	88	**91,7**	Kyphoscoliosis, Moderate OSA.
3	M	170	145	50	50	53,5	**2**	75,5	**80,2**	Severe OSA, Grade 3 Obesity, Hypoxemia, Hypercapnia, Obesity Hypoventilation Syndrome
4	F	164	119	59	44	75	**22,1**	87	**92,1**	Severe OSA, Chronic Type 1 Respiratory Failure, Grade 3 Obesity
5	F	164	93	60	34,6	42,9	**1,3**	83,8	**90,7**	Severe OSA, High-Output Cardiac Failure (B), Bilateral Positive Airway Pressure (BiPAP), Type 1 Respiratory Failure
6	M	176	130	67	42	83,4	**6,6**	68,8	**90,7**	Severe OSA, Grade 1 Obesity, Type 2 Respiratory Failure.
7	M	178	106	65	33	109,2	**25,7**	67,1	**79,2**	Severe OSA, Grade 1 Obesity, Nocturnal Hypoxemia, High-Output Positive Airway Pressure (BiPAP).
8	M	170	93	79	32	65,1	**11,1**	81,2	**91,1**	Severe OSA, Grade 3 Obesity, Night Hopyxemia, COPD (B)
9	M	177	135	71	44	29	**14**	90	**94**	Moderate OSA.
10	F	169	150	59	52,5	84,7	**18**	78	**87,3**	Severe OSA, Grade 3 Obesity, Hypoxemia, Obesity-Related Hypoventilation during Sleep.
11	F	165	150	69	55	87,2	**7,3**	74,9	**88,2**	Severe OSA, Grade 3 Obesity, Type 2 Respiratory Failure, Obesity Hypoventilation Syndrome.

12	M	177	135	62		41,6	**46,8**	87,4	**87,8**	Severe OSA, Grade 3 Obesity, Nicotine Dependence, Type 1 Respiratory Failure.
13	M	186	129	56	37	34	**7,4**	84,8	**98,6**	Severe OSA, Hypoxemia, Type 1 Respiratory Failure, Grade 2 Obesity, High-Output Positive Airway Pressure (BiPAP), Type 2 Diabetes.
14	V	184	160	51	47	108,3	**18,4**	68,7	**88,7**	Severe OSA, Grade 3 Obesity, Chronic Type 2 Respiratory Failure, Uncontrolled Persistent Bronchial Asthma, Arterial Hypertension, Chronic Heart Failure III F.C. (NYHA).

Table S2. Patient's parameters during titration

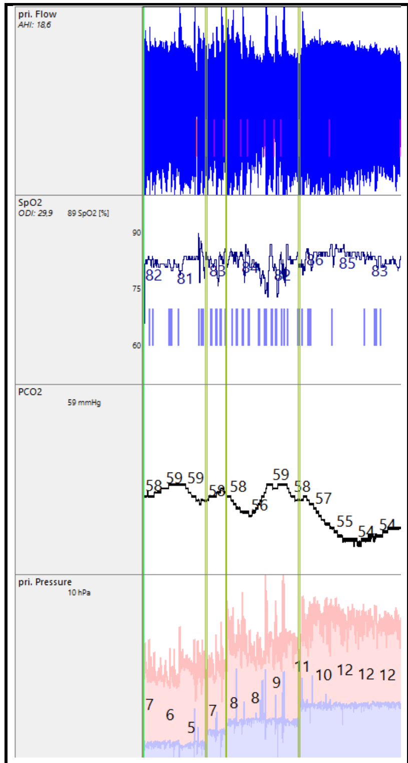	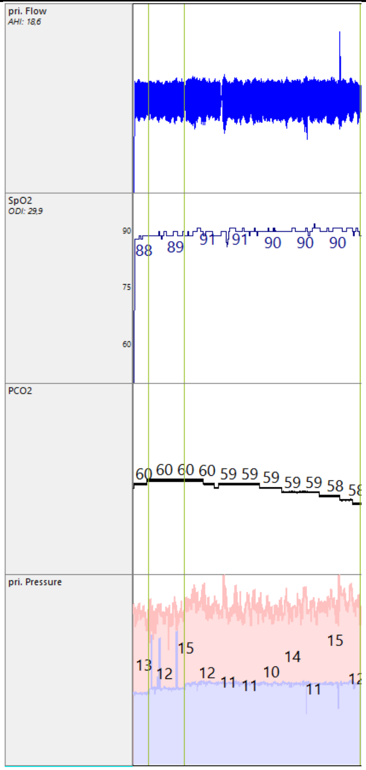	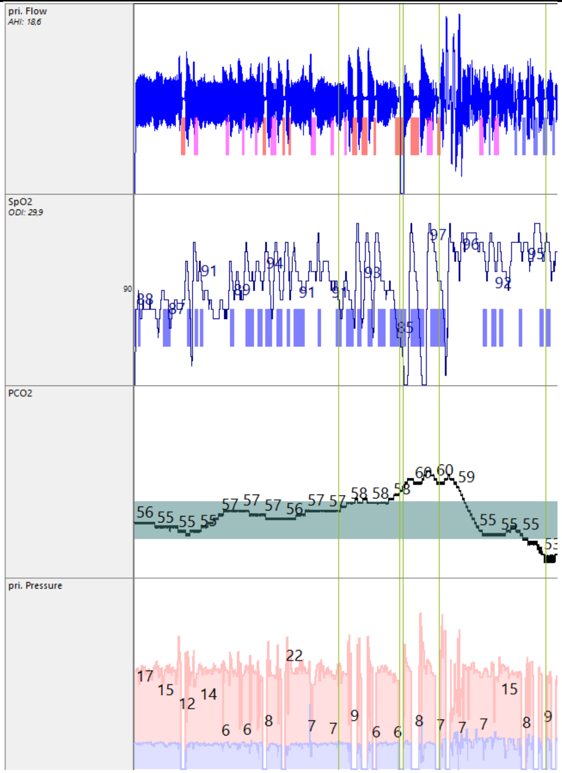
a)	b)	c)
Apnea vs CPAP pressure	pCO2 stabilized with later downtrend and additional oxygen is added	pCO2 rises with additional oxygen and then decreases when oxygen is decreases
